# Oxidative stress and inflammation combine to exacerbate cochlear damage and sensorineural hearing loss in C57BL/6 mice

**DOI:** 10.3389/fnins.2025.1563428

**Published:** 2025-03-05

**Authors:** Zhongwu Su, Yuyan Chen, Yu Liu, Jinyuan Cao, Jie Cui, Haitong Chen, Qi Li

**Affiliations:** Department of Otolaryngology, Nanfang Hospital, Southern Medical University, Guangzhou, China

**Keywords:** sensorineural hearing loss, oxidative stress, inflammation, cochlea, necroptosis, ferroptosis

## Abstract

**Background:**

Sensorineural hearing loss (SNHL) is among the most common sensory disorders, significantly affecting various aspects of the quality of life of an individual. Oxidative stress and inflammation have been involved in the progression of various forms of SNHL and are potential pathological mechanisms of the disorder. However, the synergistic effects of oxidative stress and inflammation on cochlear function is not completely understood.

**Methods:**

We explored the effects of oxidative stress and inflammation on cochlear damage and hearing impairment in male C57BL/6 mice aged 6 to 7 weeks. These in the experimental group were administered with oxidant Menadione bisulfite (MD) and the endotoxin lipopolysaccharide (LPS) via intraperitoneal route to induce oxidative stress and inflammation, whereas the control group received saline. The degree of cochlear damage was analyzed based on auditory thresholds, hair cells (HCs) loss, and the expression of protein markers related to oxidative stress, inflammation, necroptosis, and ferroptosis.

**Results:**

After six days of alternating MD and LPS injections, there was a notable elevation in hearing thresholds, which was associated with a substantial loss of HCs and spiral ganglion cells. Immunofluorescence analysis demonstrated the activation of oxidative stress, inflammation, necroptosis, and ferroptosis signaling pathways after treatment. Notably, the administration of either MD or LPS alone did not result in significant changes.

**Conclusion:**

These findings indicate that the interaction between oxidative stress and inflammation may exacerbate cochlear damage and contribute to hearing loss, potentially through the activation of necroptosis and ferroptosis pathways. Our results may identify potential therapeutic targets for the management of SNHL.

## 1 Introduction

Sensorineural hearing loss (SNHL) is recognized as one of the most prevalent sensory disorders (Géléoc and Holt, 2014). This refers to a type of hearing impairment that arises from damage to various elements of the auditory pathway, which encompasses structures from the inner ear to the auditory cortex (Wong and Ryan, 2015). This damage disrupts the function of inner ear structures. There are various forms of SNHL, including sudden deafness, age-related hearing loss (ARHL), noise-induced hearing loss (NIHL), rug-induced hearing loss, infectious hearing loss, and congenital hearing loss (Nieman and Oh, 2020). Globally, the incidence of SNHL has been steadily increasing, with approximately 1.57 billion individuals affected in 2019; projections indicate a rise to 2.45 billion individuals by 2050 (GBD 2019 Hearing Loss Collaborators, 2021). Sensorineural hearing loss significantly impacts various dimensions of an individual’s life, including emotional, psychological, social, and economic aspects (Theunissen et al., 2014). Research indicates that SNHL is a multifactorial disease influenced by various mechanisms. Recent molecular investigations have demonstrated that the initiation of SNHL is significantly associated with processes such as apoptosis, genetic mutations, autophagy, oxidative stress, and immune-inflammatory responses (Lasak et al., 2014; Cunningham and Tucci, 2017). SNHL is linked to a variety of etiological factors, with reactive oxygen species (ROS) and inflammatory cytokines playing particularly significant roles in the pathophysiology of this condition (Sagi and Stankovic, 2022).

Numerous studies have established that oxidative stress plays a substantial role in the development of SNHL (Sagi and Stankovic, 2022). The cochlea, essential for auditory functioning, exhibits increased susceptibility to oxidative stress as a result of its elevated metabolic requirements (Tan and Song, 2023). Adverse factors associated with SNHL impair the antioxidant defense mechanisms of cochlear hair cells (HCs) by inducing mitochondrial dysfunction and promoting the accumulation of ROS. This oxidative stress induces apoptosis and inflammation, resulting in irreversible cochlear degeneration and death of HCs (Maniaci et al., 2024). In ARHL, ROS production increases with advancing age, with ROS-induced damage to mitochondrial DNA (mtDNA) leading to reduced antioxidant capacity of the cochlear HCs and impairment of mitochondrial function, thereby facilitating the progression of hearing loss (Fujimoto and Yamasoba, 2014). Exposure to high levels of noise enhances ROS production in the cochlea and diminishes cochlear blood flow, which exacerbates ROS production and contributes to NIHL (Henderson et al., 2006; Maulucci et al., 2014; Shi, 2016). Clinically, ototoxic drugs such as cisplatin, carboplatin, and aminoglycoside antibiotics are established causes of irreversible hearing loss. These ototoxic substances primarily produce ROS via the apoptotic pathway, resulting in damage to HCs within the organ of Corti (Jiang et al., 2017; Sheth et al., 2017). Notably, various studies have demonstrated that antioxidants, including glutathione, cysteine, vitamin E, D-methionine, and methionine, alleviate oxidative damage caused by ROS, reduce mtDNA mutations in animal models, and improve hearing outcomes (Seidman and Babu, 2003; Lynch and Kil, 2005; Heman-Ackah et al., 2010; Oishi and Schacht, 2011; Delhez et al., 2020). The accumulation of ROS in the cochlea triggers the release of inflammatory cytokines, which further exacerbate cochlear damage (Sagi and Stankovic, 2022).

Previous studies have demonstrated that immune and inflammatory responses play significant roles in various forms of SNHL, with anti-inflammatory therapies proving effective in restoring hearing. Research has established an association between systemic inflammation and age-related diseases, revealing inflammatory and immune responses as critical mechanisms in the onset and progression of ARHL (Shi, 2016). Similarly, in NIHL, exposure to noise can trigger an inflammatory response, leading to elevated levels of inflammatory mediators (Mogi et al., 1982). Additionally, the pathophysiology of sudden sensorineural hearing loss is associated with viral infections and immune-mediated mechanisms (Mogi et al., 1985). The immunomodulatory effects of corticosteroids have been extensively investigated and they remain the primary treatment for SNHL (Spear and Schwartz, 2011). A prospective, randomized controlled trial comprising 116 patients with rapidly progressive bilateral SNHL found that 57% of patients under a one-month course of corticosteroid therapy experienced improved hearing outcomes (Zeitoun et al., 2005).

Numerous studies have demonstrated that oxidative stress and inflammation are intricately linked to the progression of various forms of SNHL and the subsequent damage to the inner ear structures, which represent common pathological features (Mathews and Kumar, 2003). However, the synergistic effects of inflammation and oxidative stress on the cochlea remain incompletely understood. In this study, we used the oxidant menadione bisulfite (MD) (Ossola et al., 2000) and endotoxin lipopolysaccharide (LPS) to simultaneously induce oxidative stress and inflammation. We then assessed auditory function, HC integrity, and spiral ganglion cell viability after treatment. Additionally, we investigated the potential cell death pathways.

## 2 Materials and methods

### 2.1 Animals

Preliminary experiments were conducted across three independent batches to optimize the drug concentration for inducing SNHL. The three pilot batches collectively comprised 11 experimental cohorts (drug-treated) and 11 matched control cohorts, with three mice allocated to each group, yielding a total sample size of 66 mice. In the definitive experimental phase, twenty-four male C57BL/6 mice (6–7 weeks old, 18–21 g) were housed in a specific pathogen-free (SPF) facility at the Experimental Animal Center of Southern Medical University (Guangzhou, China). Animals were maintained on a 12-h light/dark cycle under controlled temperature (20–26°C) and humidity (40–70%) conditions. Food and water were provided ad libitum. All experimental procedures were approved by the Animal Ethics Committee of Southern Medical University (approval number SMUL202311016).

### 2.2 Experimental procedure

**Figure 1:**
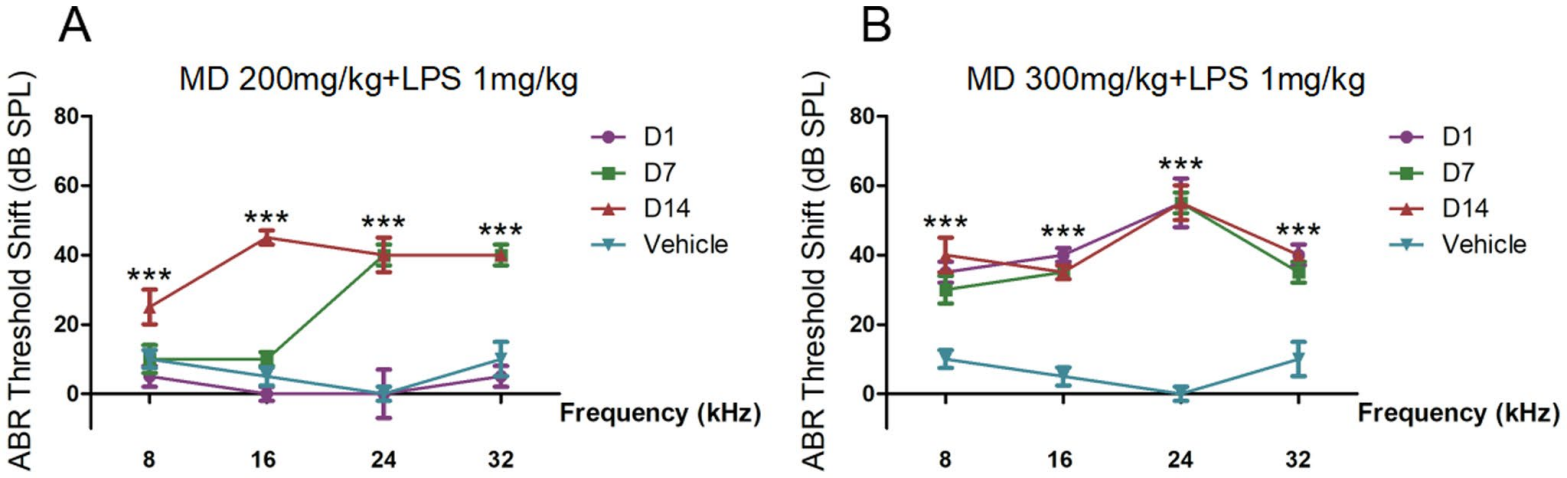
The auditory brainstem response (ABR) thresholds shift of preliminary experiments. **(A)** BR threshold shifts across 8, 16, 24, and 32 kHz in C57BL/6 mice treated with MD 200 mg/kg and LPS 1 mg/kg every other day for 9 days measured at 1-, 7-, and 14-day post-treatment intervals. **(B)** ABR threshold shifts across 8, 16, 24, and 32 kHz in C57BL/6 mice treated with MD 300 mg/kg and LPS 1 mg/kg every other day for 9 days measured at 1-, 7-, and 14-day post-treatment intervals. Statistical significance was determined by one-way ANOVA. *p < 0.05; **p < 0.01; ***p < 0.001; ns: not significant.

Preliminary experiments conducted to investigate the effects of varying drug concentrations revealed that the administration of different doses of MD (25, 50, 100, 150, 200, 300, and 400 mg/kg) through continuous intraperitoneal (i.p.) injection over a period of 10 days did not lead to a statistically significant elevation in hearing thresholds.(Osswald et al., 1987; Ossola et al., 2000; Ahmad and Srivastava, 2007; Amiti et al., 2019). The administration of a combination of MD (300 mg/kg) and LPS (2 mg/kg) (Ma et al., 2022) via i.p. injection over a period of five days led to the mortality of all experimental subjects, suggesting that the dosage was excessively high and beyond tolerable limits. A reduction in dosage to MD 100, 200, and 300 mg/kg, in conjunction with LPS administered at a dose of 1 mg/kg every other day over a period of 9 days, resulted in observable changes in threshold shifts within the high-dose group (Figure 1). In order to enhance the model’s efficacy, we established the drug concentrations at MD 300 mg/kg and LPS 1 mg/kg, with both agents being administered intraperitoneally on an alternate-day schedule over a duration of six days.

**Figure 2:**
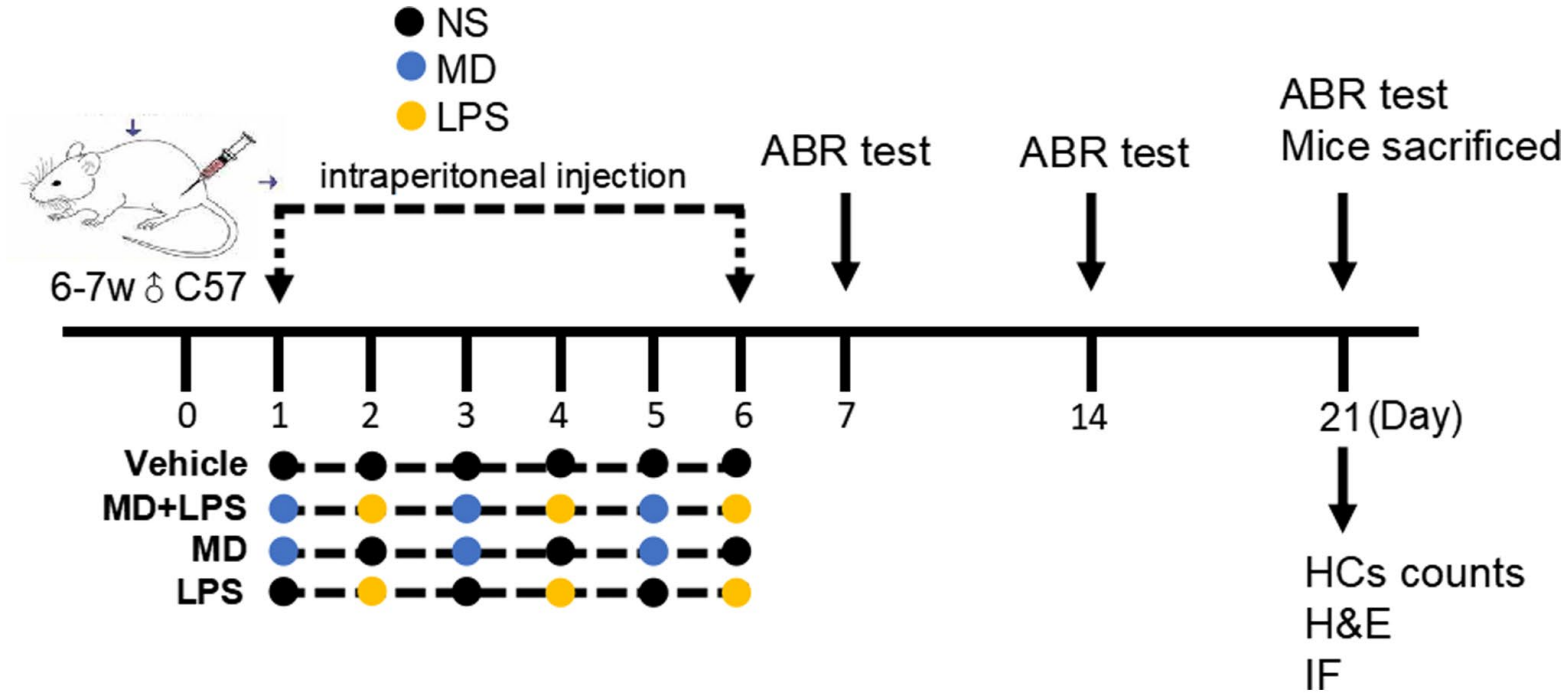
Experimental procedure of the administration of MD and LPS treatments in C57BL/6 mice. The baseline ABR of mice was measured before treatment. Mice were administered an intraperitoneal injection of vehicle, MD only, LPS only, or a combination of MD and LPS on alternate days for six days. Subsequently, ABR thresholds were recorded at 1d, 7d and 14d after the administration. After the completion of third ABR assessment, all the mice were sacrificed to evaluate the extent of cochlear damage.

The experimental procedure was comprised of a total of 24 male C57BL/6 mice (Figure 2). Prior to the treatment, baseline ABR testing was conducted on all mice to assess normal hearing, functional auricular reflex, and unobstructed external auditory canals. To induce acute oxidative stress, MD was used, while LPS was used to induce inflammation. After application of toe markings, the mice were randomly assigned into four groups: vehicle, MD + LPS, MD, and LPS, with each group comprising six mice. Mice in the MD + LPS group received alternating i.p. injections of MD (300 mg/kg; MedChemExpress, USA, Cat# HY-B1897A) on days 1, 3, and 5, and LPS (1 mg/kg, MedChemExpress, Cat# HY-D1056C2) on days 2, 4, and 6. Mice in the MD group were administered three i.p. injections of MD on days 1, 3, and 5, while receiving three i.p. injections of normal saline (NS) on days 2, 4, and 6. Conversely, the LPS group received LPS injections (days 2, 4, 6) interspersed with NS administration (days 1, 3, 5). The vehicle group received an equal volume of solvent via i.p. injections once daily for 6 days. Following the 6-day i.p. drug administration protocol, one mouse in the MD + LPS group was dead, likely attributable to i.p. administration intolerance. The remaining four experimental groups showed good condition in intake and activity throughout the study period. Longitudinal body weight measurements for all groups are documented in Supplementary Figure 1.

### 2.3 Auditory brainstem response (ABR)

Auditory function was evaluated through the measurement of ABR utilizing a Tucker-Davis Technologies System 3 (TDT 3, Alachua, FL, USA), following the methodology outlined by Su et al. (2024). The ABR testing was performed on all the 24 mice at baseline and on days 1, 7, and 14 after treatment. Before the commencement of testing, the mice were subjected to anesthesia by i.p. injection of 1% pentobarbital sodium (50 mg/kg) and positioned on a heating pad to maintain their body temperature. Subsequently, subdermal needle electrodes were strategically placed at the vertex of the skull to serve as the active electrode, while a reference electrode was positioned beneath the left ear, and a ground electrode was located in the right ear. The ABR responses were recorded at frequencies of 8, 16, 24, and 32 kHz. The mean response to 1,024 stimuli was documented by systematically reducing the sound intensity in increments of 5 dB as it approached the threshold. The threshold was defined as the lowest intensity at which a positive wave could be detected. The sound intensity was systematically reduced from 80 dB SPL to 5 dB SPL in increments of 5 dB until the characteristic brain wave was no longer detectable. The threshold was determined as the lowest stimulus level where a positive wave was evident. All ABR measurements were conducted by the same experimenter in a standard acoustic chamber to ensure consistency and reliability of the results.

### 2.4 Tissue preparation

Following terminal ABR assessment on 21 days post-treatment initiation, mice were euthanized via pentobarbital sodium (50 mg/kg, i.p.) and decapitation. For cochlear cryosectioning, temporal bones were meticulously dissected to reveal the cochleae. The cochleae were subsequently preserved in a solution of 4% paraformaldehyde (PFA) (Boster Biology Technology, China, AR0030) in 0.01 M phosphate-buffered saline (PBS) at a temperature of 4°C overnight. Following fixation, the cochleae underwent in a 10% solution of sodium ethylenediaminetetraacetic acid (pH 7.4) for three days, followed by incubation in gradient sucrose solutions (10, 20, and 30%) for 24 h each. The cochleae were then embedded in optimal cutting temperature (OCT) compound and sectioned along the cochlear axis using a cryostat (Thermo, USA). Cryosections were cut to a thickness of 10 μm and subsequently preserved at −80°C for subsequent analysis.

### 2.5 Immunofluorescence of cochlear cryosections

Immunofluorescence procedures were performed in accordance with the methodologies outlined in our previous study (Xiong et al., 2019). In summary, cochlea sections were subjected to an initial incubation in a 3% Triton X-100 solution (Sigma-Aldrich, USA, T8787) in PBS at room temperature for a duration of 30 min. Additionally, the sections were incubated with a 5% goat serum solution (Solarbio, China, SL038) in PBS for one hour at ambient temperature, subsequently followed by three washes, each lasting five minutes with PBS. Subsequently, in a diluent at 4°C for 48 h, the cochlea samples were subjected to incubation with primary antibodies, including anti-4-HNE (1:200, Abcam, UK, ab48506, mouse), anti-IL-1β (1:50, Abcam, AB254360, rabbit), anti-IL-1R1 (1:100, Proteintech Group, USA, #27348-1-AP, rabbit), anti-pNF-κB (1:100, Cell Signaling Technology, USA, #3033T, rabbit), anti-P-RIPK3 (1:100, Cell Signaling Technology, #91702, rabbit), anti-RIPK3 (1:50, Cell Signaling Technology, #95702, rabbit), anti-MLKL (1:100, Proteintech Group, #66675-1-1g, mouse), anti-GPX4 (1:  100, Abcam, ab125066, rabbit), anti-TNF-*α* (1:200, Proteintech Group, #17590-1-AP, rabbit), anti-iNOS (1:100, Cell Signaling Technology, #13120, rabbit). After thorough washing with PBS, the sections were incubated with Alexa Fluor 594-conjugated donkey anti-mouse IgG (1:  200, Invitrogen, A21203), Alexa Fluor 594-conjugated donkey anti-rabbit IgG (1:  200, Invitrogen, A21206) and Alexa Fluor 488-conjugated donkey anti-mouse IgG (1:  200, Invitrogen, A21202) at a temperature of 4°C overnight, in a dark environment. After further washes with PBS, the sections underwent counterstaining with DAPI (1:  1000, Solarbio, S2110) for 30 min at room temperature. Subsequently, the cochlear samples were examined using a NIKON Confocal Microscope (Nikon-Eclipse-Ti, Japan). Fluorescence quantification was performed by measuring mean intensity in 20 × 20 μm^2^ ROIs (3 regions/sample) using ImageJ FIJI (v2.3.0), with cochlear sections oriented using Reissner’s membrane as anatomical landmark in accordance with AIMQ guidelines for reproducible microscopy (Schmied et al., 2024). Relative fluorescence intensity was calculated as [(Experimental group mean fluorescence) / (Vehicle-treated control mean)] × 100%, normalized to DAPI counterstain (Solarbio, S2110).

### 2.6 Cochlear surface preparations

Randomly selected cochleae (*n* = 3 per group) were dissected immediately after euthanasia via cervical dislocation under pentobarbital anesthesia (50 mg/kg, i.p.). The temporal bones were rapidly excised and immersed in 4% PFA/PBS (Boster Biology Technology, China, AR0030) for 24 h at 4°C. Following three-day decalcification in 10% ethylenediaminetetraacetic acid (pH 7.4), the specimens underwent meticulous microdissection to remove surrounding osseous and membranous tissues, ultimately exposing intact Corti organs for morphological analysis. Then, we cut the auditory epithelium into three turns (the apex, middle, base). The cochlear tissues were subjected to permeabilization using a 0.3% Triton X-100 for 30 min at room temperature. After blocking with a 5% goat serum solution for one hour at ambient temperature, the specimens were incubated with Myosin VIIa antibody (1:100, Bioss, China, #bs-7761R-FITC, rabbit) at 4°C overnight to visualize hair cells. After 3 washes with PBS, the specimens were stained with Alexa Fluor 594-conjugated donkey anti-rabbit IgG (Invitrogen, A21206) for 1 h in the dark at room temperature. Then the specimens were washed and counterstained with DAPI (1:1000, Solarbio, S2110) as described above in the dark for 30 min at room temperature, and examined using a NIKON Confocal Microscope (Nikon-Eclipse-Ti, Japan).

### 2.7 Hair cell counts

Subsequent to the completion of the third ABR test, we acquired cochlear and prepared cochlear surface preparations as described above. Images were captured by a scale bar of 50 μm with a NIKON confocal microscope (Nikon-Eclipse-Ti, Japan). Hair cells were counted from the apex to the base in order to determine the rate of loss. Subsequently, the percentage of hair cell loss was calculated utilizing a cytocochleogram as described by Müller’s,as analyzed by two-way ANOVA (Viberg and Canlon, 2004).

### 2.8 Hematoxylin–Eosin (H&E) staining

The 10-μm cochlear sections as described above were initially washed twice with PBS. The sections were then mounted on slides and subjected to hematoxylin staining for a duration of 10 min, after which they were rinsed in running water for 5 min. The cochlear sections underwent counterstaining with eosin for a duration of three minutes. After counterstaining, the slides were dehydrated: by sequentially immersing in 80, 90, and 95% ethanol for 10 s each, and 100% ethanol for 1 min. The slides were then transferred to a fume hood and immersed in xylene solution I for 5 min to enhance full transparency. Following these steps, the slides were then transferred to xylene solution II for an additional 5 min. The slides were then allowed to air dry for 20 min to eliminate any residual xylene, after which they were sealed using neutral resin. The prepared slides were scanned using an optical microscope (Aperio Versa8, LEICA).

### 2.9 Statistical analysis

Each group of mice was randomly assigned and each staining experiment was replicated at least three times. Descriptive statistics are expressed as means ± SD and analyzed using SPSS software and GraphPad Prism 8 software. To assess differences between two groups, a two-tailed Student’s *t*-test was employed for pairwise comparisons, while a one-way analysis of variance (ANOVA), accompanied by Tukey’s *post hoc* test, was utilized for comparisons among multiple groups. For ABR threshold analysis across the four groups, two-way analysis of variance (ANOVA) with Sidak’s post hoc test was performed. A *p*-value of less than 0.05 was deemed statistically significant.

## 3 Results

### 3.1 The combination of MD and LPS promotes hearing loss in C57BL/6 mice

**Figure 3:**
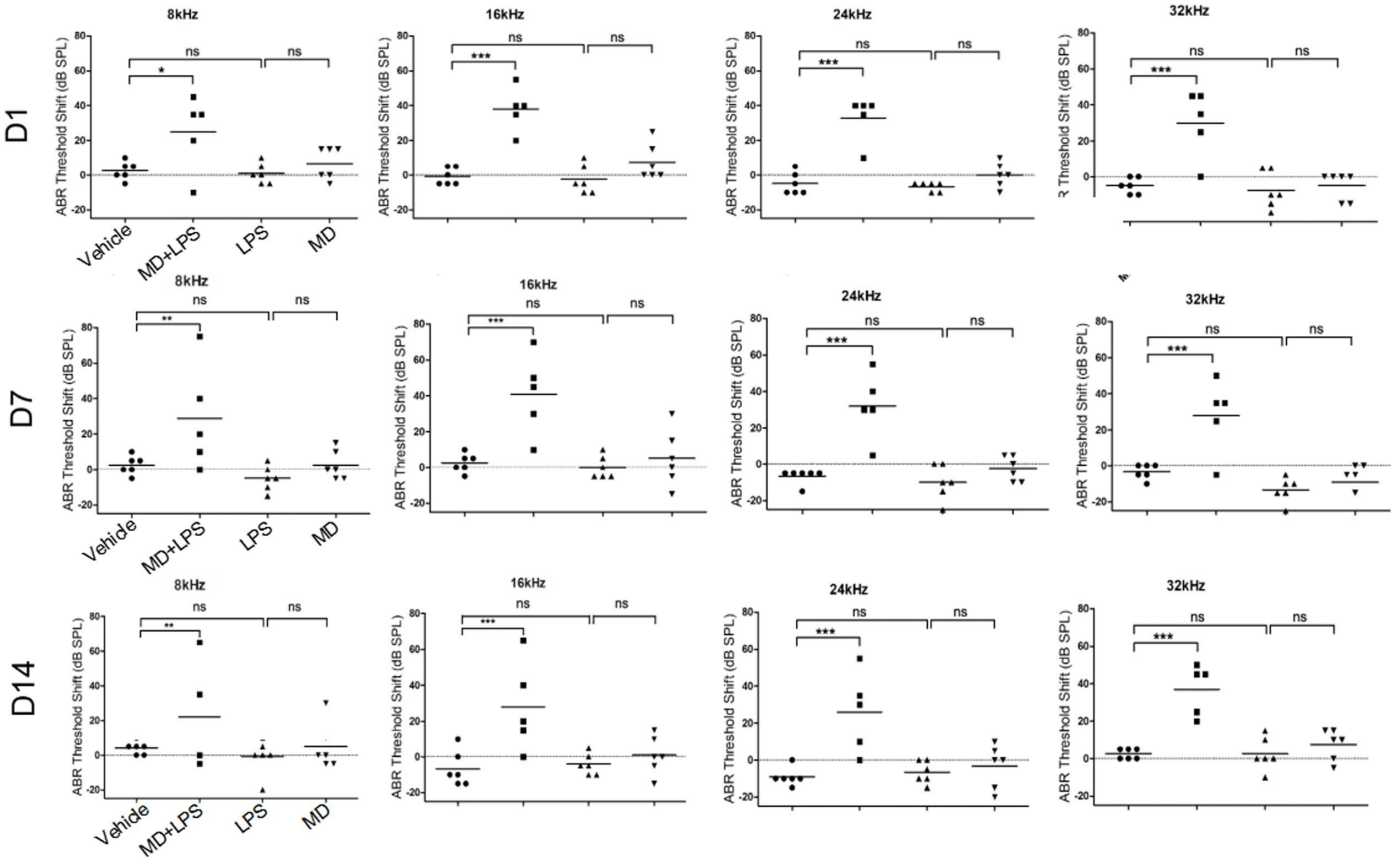
The combination of MD and LPS promotes hearing loss in C57BL/6 mice. ABR threshold shifts across 8, 16, 24, and 32 kHz in C57BL/6 mice treated with MD, LPS, or MD + LPS combination, measured at 1-, 7-, and 14-day post-treatment intervals. The data are shown as individual points and the means ± SD of each group. Statistical significance was determined by two-way ANOVA. *p < 0.05; **p < 0.01; ***p < 0.001; ns: not significant.

To assess the impact of MD and LPS on auditory function, ABR threshold was assessed 3 times in the four groups of mice over a period of 14 days following administration (Vehicle, MD+LPS, MD, LPS). The ABR thresholds were assessed at frequencies of 8, 16, 24, and 32 kHz, as shown in Figure 3. Prior to administration, no significant differences in ABR thresholds were observed between the experimental group and the control group. The average ABR thresholds shift in the MD + LPS group (*n* = 5) were significantly increased on day 1 after the 6 days of i.p. drug administration (35.00 ± 19.69 dB at 8 kHz, 46.00 ± 13.87 dB at 16 kHz, 61.00 ± 14.75 dB at 24 kHz, and 67.00 ± 15.65 dB at 32 kHz) relative to the control group (*n* = 6), as analyzed by two-way ANOVA. However, one mice did not exhibit significant changes at any frequency compared to the baseline characteristics, indicating individual differences variability and suggesting that some mice might resist the effects of both drugs. On day 7, the ABR thresholds shift in the MD + LPS group remained stable compared to day 1 post-administration (39.00 ± 25.35 dB at 8 kHz, 49.00 ± 21.01 dB at 16 kHz, 60.00 ± 18.37 dB at 24 kHz, and 65.00 ± 17.32 dB at 32 kHz). Although the ABR thresholds shift in the MD + LPS group minimally decreased on day 14, significant differences were still observed compared to pre-administration levels (32.00 ± 24.14 dB at 8 kHz, 36.00 ± 24.08 dB at 16 kHz, 54.00 ± 20.74 dB at 24 kHz, and 74.00 ± 8.94 dB at 32 kHz). Conversely, no notable differences in ABR thresholds shift were detected in the MD group (*n* = 6), or LPS group (*n* = 6) on days 1, 7, or 14 post-administration. The average ABR thresholds of mice in four groups both prior and following administration, were presented in Supplementary Tables 1–4. The results of this study suggest the combination of 300 mg/kg MD and 1 mg/kg LPS, administered intraperitoneally every other day for 6 days, resulted in permanent hearing impairment, with the overall hearing threshold shift of mice increasing. However, no significant changes were observed in the MD or LPS groups compared to their baseline thresholds. These results indicated that the combination of MD and LPS promotes hearing loss in mice.

### 3.2 The combination of MD and LPS induces the loss of HCs and spiral ganglion cells

**Figure 4:**
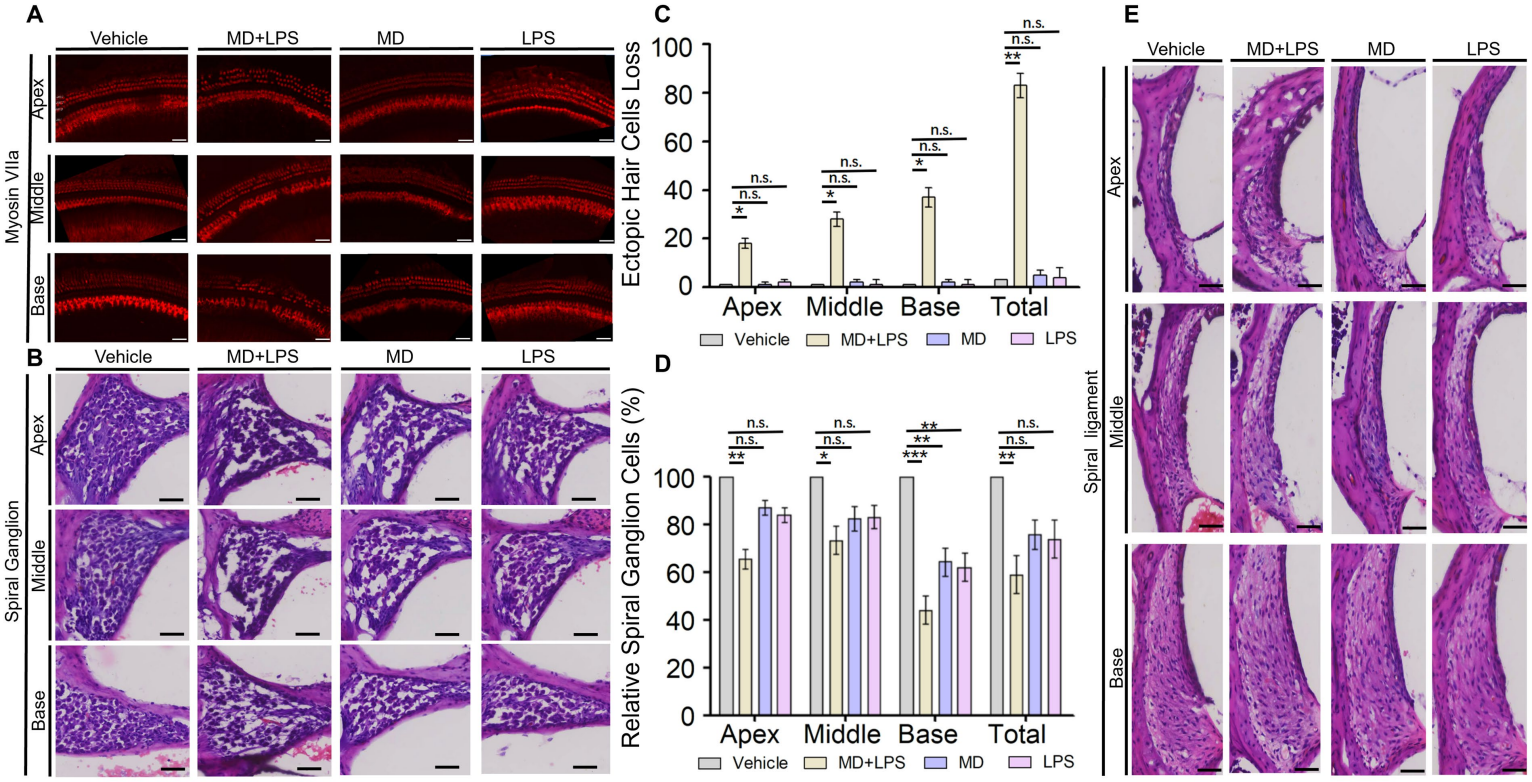
The combination of MD and LPS induces the loss of HCs and spiral ganglion cells. **(A)** Immunolabeling of the HC marker myosin VIIa. Confocal microscopy was employed to capture images of whole mounts of HCs in the cochlea (three mice per subgroup). *n* = 3 for each condition. Scale bar: 50 μm. **(B)** Representative H&E staining images of spiral ganglion cells in the cochlea for each group at 14 days post-drug administration (three miceper subgroup). *n* = 3 for each condition. Scale bar: 50 μm. **(C)** The numbers of three turns and overall ectopic HCs loss in the four groups of cochlea. *n* = 3 for each condition. Statistical significance was determined by two-way ANOVA. **(D)** Relative spiral ganglion cells of cochlea in four groups compared to the control group (%). *n* = 3 for each condition. Statistical significance was determined by two-way ANOVA. **(E)** Representative images of H&E staining displaying the vascular lines and spiral ligament of the cochlea (three mice per subgroup). *n* = 3 for each condition. Scale bar: 50 μm. *p < < 0.05; **p < < 0.01; ***p < < 0.001; ns, not significant.

To investigate the morphological and functional changes in the cochlea on day 14 post-administration, we examined the alterations in the number of cochlear HCs by performing immunolabeling of the HCs marker myosin VII and H-E staining on frozen cochlear sections. The results showed that in the MD + LPS group, a significant reduction in outer hair cells (OHCs) was observed in the apex turn of the cochlear when compared to the control group. Additionally, there was a notable loss of both inner hair cells (IHCs) and OHCs in the middle and basal turns, with the loss of hair cells in the basal turn being especially pronounced. Meanwhile, the structure and organization of HCs showed a significant loss and disorganized arrangement compared to the control group (Figures 4A,C). Conversely, a minimal reduction in HC numbers was observed in the MD and LPS groups, with IHCs and OHCs remaining well-organized and structurally intact observing under a microscope. Additionally, spiral ganglion cells, crucial for auditory signal transmission and the precision of auditory function, were affected. The H-E staining analysis revealed a significant reduction in the density of cochlear spiral ganglion cells in the MD + LPS group compared to the control group (Figures 4B,D). While a minimal reduction in spiral ganglion cell density was observed in the MD and LPS groups, this change was significantly less compared to the MD + LPS group. Furthermore, there were not significant morphological alterations in the cochlear vascular structures or spiral ligament between the experimental and control groups (Figure 4E). These morphological alterations indicate that the combination of MD and LPS contributes to the loss of HCs and spiral ganglion cells, subsequently impairing auditory function.

### 3.3 Activation of oxidative stress and inflammatory signaling in the cochlea

**Figure 5:**
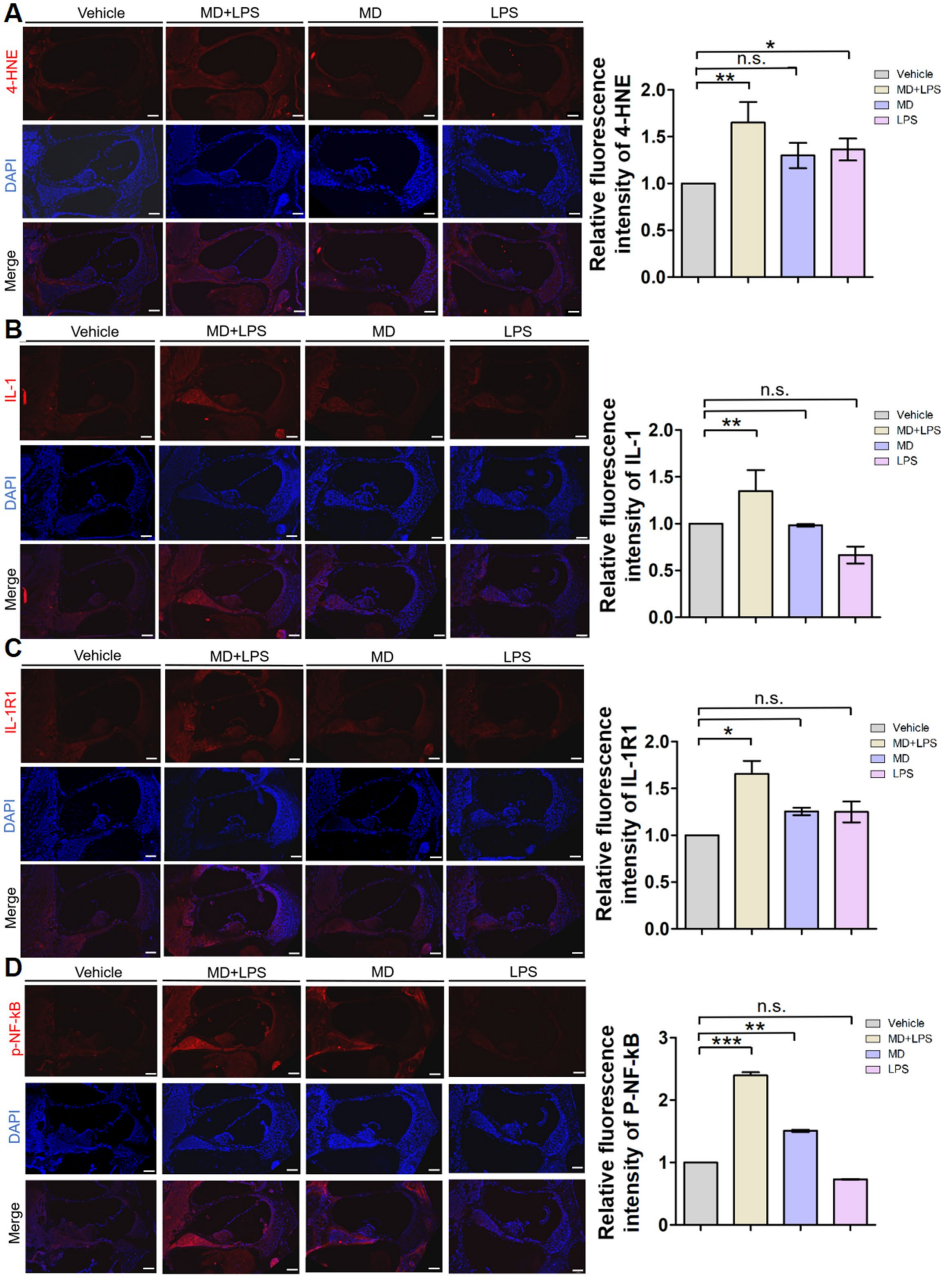
Oxidative stress and inflammatory signals are activated in cochlear sections after administration. **(A)** Representative 4-HNE immunofluorescence staining images in the cochlea. The 10 μm frozen sections of cochlea from the MD + LPS, MD, and LPS groups (three mice per subgroup) show enhanced 4-HNE (red) compared to the control mice. Relative fluorescence intensity of 4-HNE. *n* = 3 for each condition. **(B)** Representative IL-1 immunofluorescence staining images in the cochlea. The frozen sections of the cochlea in the MD + LPS group show enhanced IL-1 (red) compared to the other groups. Relative fluorescence intensity of IL-1. *n* = 3 for each condition. **(C)** Representative IL-1R1 immunofluorescence staining images in the cochlea. The frozen sections of the cochlea in the MD + LPS and LPS groups show enhanced IL-1R1 (red) compared to the other groups. Relative fluorescence intensity of IL-1R1. *n* = 3 for each condition. **(D)** Representative p-NF-kB immunofluorescence staining images in the cochlea. The frozen sections of the cochlea in the MD + LPS and MD groups show enhanced p-NF-kB (red) compared to the other groups. Relative fluorescence intensity of p-NF-kB. *n* = 3 for each condition. The pictures above were taken from the base turn of the cochlea. Sections were counterstained with DAPI (blue) to highlight the nuclei. Scale bar: 100 μm. Statistical significance was determined by two-way ANOVA. *p < 0.05; **p < 0.01; ***p < 0.001; ns: not significant. DAPI, 4′,6-diamidino-2-phenylindole.

To investigate whether oxidative stress and inflammatory signaling pathways are activated in the cochlea of mice following treatment with MD and LPS, we conducted immunofluorescence staining to determine oxidative stress and inflammatory markers in cochlear sections from C57BL/6 mice treated with vehicle, MD, LPS, or a combination of MD and LPS. Results of the immunofluorescent analysis demonstrated a significant increase in the levels of the oxidative stress marker 4-HNE and inflammatory markers IL-1, IL-1R1, and p-NF-κB in the MD + LPS group compared to the vehicle controls (Figure 5), indicating the upregulation of oxidative stress and inflammatory signaling pathways. In the MD group, the expression of 4-HNE, IL-1R1, and p-NF-κB was moderately elevated compared to the vehicle controls. Similarly, the expression of 4-HNE and IL-1R1 was increased in the LPS group. In addition, the inflammatory markers inducible nitric oxide synthase (iNOS) and tumor necrosis factor-*α* (TNF-α) were found to be elevated in both the MD + LPS and LPS groups (Supplementary Figure 2). Furthermore, the introduction of the MD drug in the MD + LPS group exacerbated the inflammatory response. These findings indicate that both oxidative stress and inflammatory pathways are activated following MD and LPS administration.

### 3.4 Activation of necroptosis and ferroptosis pathways in C57BL/6 mice

**Figure 6:**
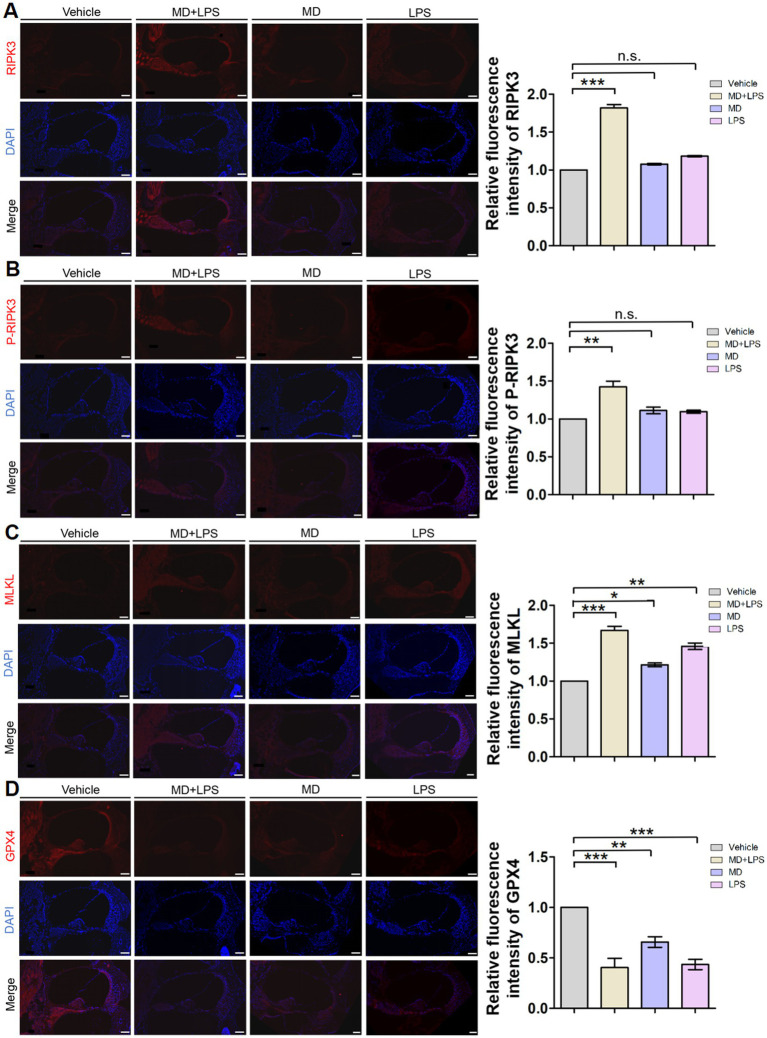
Necroptosis and ferroptosis signaling pathways are activated in cochlear sections after administration. **(A)** Representative RIPK3 immunofluorescence staining images in the cochlea. The 10 μm frozen sections of cochlea from the MD + LPS group (three mice per subgroup) show enhanced RIPK3 (red) compared to the vehicle controls. Relative fluorescence intensity of RIPK3. *n* = 3 for each condition. **(B)** Representative P-RIPK3 immunofluorescence staining images in the cochlea. The frozen sections of the cochlea in the MD + LPS group show enhanced P-RIPK3 (red) compared to the other groups. Relative fluorescence intensity of P-RIPK3. *n* = 3 for each condition. **(C)** Representative MLKL immunofluorescence staining images in the cochlea. The frozen sections of cochlea in the MD + LPS, MD and LPS groups show enhanced MLKL (red) compared to the other groups. Relative fluorescence intensity of MLKL. *n* = 3 for each condition. **(D)** Representative GPX4 immunofluorescence staining images in the cochlea. The frozen sections of cochlea in the MD + LPS, MD, and LPS groups show decreased GPX4 (red) compared to the vehicle controls. Relative fluorescence intensity of GPX4. *n* = 3 for each condition.

Previous studies have demonstrated that various cell death pathways are associated with HC loss and SNHL. Necroptosis and ferroptosis are forms of regulated cell death, which have been implicated in SNHL. To investigate the relationship between these cell death pathways and oxidative stress and inflammation, we performed immunofluorescence staining of the relevant markers. Immunofluorescent analysis revealed a significant elevation in the necroptosis markers RIPK3, P-RIPK3, and MLKL in the MD + LPS group compared to vehicle controls, with the most significant increase observed in RIPK3 (Figure 6). The fluorescence intensities of these markers were enhanced in the spiral ligament, vascular structures, cochlear structures, and spiral ganglion. Furthermore, the expression of MLKL was slightly increased in the MD and LPS group. Nonetheless, no substantial differences were observed in the immunofluorescence intensity of these markers between the MD, LPS, and control groups. Immunofluorescence staining of the ferroptosis marker glutathione peroxidase 4 (GPX4) revealed a significant decrease in expression levels in the MD + LPS, MD, and LPS groups compared to the vehicle controls. The most pronounced reduction was observed in the MD + LPS group, indicating that oxidative stress and inflammation trigger ferroptosis. Collectively, these results indicate that the combination of oxidative stress and inflammation is associated with the activation of necroptosis and ferroptosis pathways.

## 4 Discussion

Hearing loss constitutes a major public health concern on a global scale, involving complex molecular pathways. Increasing evidence suggests that oxidative stress and inflammation are involved in processes of various forms of SNHL and are likely to be common pathological features. However, the combined effects of oxidative stress and inflammation on cochlear function remain incompletely understood. In this study, we demonstrate that the combination of oxidative stress and inflammation exacerbates cochlear damage, leading to significant hearing impairment.

In this study, we performed a comparative analysis of hearing function, morphological changes, and immunofluorescent staining among four groups of mice. Our results reveal that the combination of MD and LPS results in substantial and permanent hearing loss. Conversely, neither MD nor LPS alone caused a significant increase in the hearing threshold. This observation aligns with those of the research conducted by Ji-Hyun et al., who developed a systemic LPS injection model that could induce hearing loss in NLRP3 mutant mice, while control mice did not exhibit such effects, validating the relevance of our model (Ma et al., 2022). Notably, throughout this study, no substantial cochlear damage or hearing loss was observed with MD or LPS treatment alone. However, prolonged exposure or extended observation periods could reveal potential effects on hearing. These findings indicate that the combination of oxidative stress and inflammation exacerbates cochlear damage and contributes to hearing loss.

Oxidative stress in the cochlea triggers an inflammatory response, resulting in the stimulation of immune cells (Sagi and Stankovic, 2022). Reactive oxygen species activate inflammatory pathways, including mitogen-activated protein kinase (MAPK) and nuclear factor-κB (NF-κB) signaling cascades, resulting in the production of pro-inflammatory cytokines, adhesion molecules, and chemokines (Tan et al., 2016; Ma et al., 2022). These inflammatory mediators extend the inflammatory response, exacerbating HC loss and hearing impairment (Fujioka et al., 2006; Okano et al., 2008). The findings of this study indicate increased expression of inflammatory mediators such as IL-1, IL-1R1, and p-NF-κB in the MD group, compared to the vehicle controls, indicating that oxidative stress induces inflammation. Additionally, activated immune cells produce increased levels of ROS, thereby amplifying a feedback loop that exacerbates cochlear damage (Reuter et al., 2010; Kalinec et al., 2017; Prasad, 2017). Immunofluorescent analysis revealed upregulation of the oxidative stress marker 4-HNE in the LPS group, compared to the vehicle controls, indicating that inflammation induces oxidative stress. These findings indicate that oxidative stress and inflammation may interact synergistically, establishing a self-perpetuating and detrimental cycle.

Ototoxic drugs, noise exposure, and aging are recognized pathogenic factors contributing to hearing loss, leading to auditory damage and the degeneration of HCs. Unfortunately, cochlear HCs in mammals are non-regenerative (Rai et al., 2021; Gill et al., 2024). Research has demonstrated that various cell death pathways are implicated in HC loss, including necroptosis and ferroptosis (Dixon et al., 2014; Yang et al., 2014; Fulda, 2016; Su et al., 2024). Here, we found that the combination of MD and LPS led to the degeneration of HCs and spiral ganglion cells. Additionally, we detected a significant elevation in markers of necroptosis in the cochlea, alongside a significant decrease in the ferroptosis marker GPX4 and an increase in the expression of 4-HNE after the combined treatment of MD and LPS. Several studies have investigated the mitochondrial stress responses in SH-SY5Y cells, the most commonly used neuronal cell line, when subjected to paraquat exposure. These responses are associated with multiple cell death pathways, including necroptosis, ferroptosis, and autophagy (Hirayama et al., 2018). In a sepsis mouse model, exposure to LPS triggered cardiomyocyte contractile dysfunction, oxygen molecules accumulation, apoptosis, necroptosis, and ferroptosis. These pathological responses were mitigated by the administration of oleanolic acid, a mitophagy inducer, as well as through the inhibition of ACSL4 and ferroptosis (Li et al., 2024). These findings suggest that cochlear damage induced by the combined effects of oxidative stress and inflammation is associated with necroptosis and ferroptosis.

Our study is the first time to utilize MD and LPS to induce oxidative stress and inflammation via i.p. injection. The findings suggest that oxidative stress and inflammation may intensify cochlear damage and play a role in the development of hearing loss. It is important to note that the administration of either MD or LPS in isolation did not produce any significant alterations. This may establish a basis for future investigations into the mechanisms and therapeutic approaches for SNHL. Meanwhile, there are some limitations in our experiment. First of all, the administration of drugs via i.p. injection in the context of MD and LPS may result in adverse effects on various organs and systems, as the substances are absorbed into the bloodstream through the abdominal veins. We conducted an investigation to identify the optimal drug concentration by implementing a series of continuous dosing trials. During this process, we monitored the mice’s weight, dietary intake, and activity levels to mitigate the extent of systemic damage. Secondly, we need to further investigate how MD and LPS drugs adversely affect the cochlear spiral ganglion cells, hair cells, resulting in auditory impairment. Finally, our immunofluorescence analysis revealed the activation of necroptosis and ferroptosis in the MD + LPS group. This finding indicates a potential association between oxidative stress and inflammation with the activation of these cell death pathways. However, the precise mechanisms and signaling pathways involved warrant further investigation.

## 5 Summary and conclusion

In conclusion, we used a combination of MD and LPS to induce oxidative stress and inflammation in C57BL/6 mice. The combination of these two agents exacerbated cochlear damage, activated oxidative stress and inflammatory pathways, and might be related to the activation of necroptosis and ferroptosis. However, single administration of either drug exhibited no significant changes. Our findings highlight potential mechanisms underlying cochlear damage in SNHL and may offer novel insights for the development of effective prevention and treatment strategies.

## Data Availability

The raw data supporting the conclusions of this article will be made available by the authors, without undue reservation.
