# Correction: Oxidative stress and inflammation combine to exacerbate cochlear damage and sensorineural hearing loss in C57BL/6 mice

**DOI:** 10.3389/fnins.2025.1640349

**Published:** 2025-07-29

**Authors:** Zhongwu Su, Yuyan Chen, Yu Liu, Jinyuan Cao, Jie Cui, Haitong Chen, Qi Li

**Affiliations:** Department of Otolaryngology, Nanfang Hospital, Southern Medical University, Guangzhou, China

**Keywords:** sensorineural hearing loss, oxidative stress, inflammation, cochlea, necroptosis, ferroptosis

There was a mistake in Figure 4 as published. During the final assembly of the figure panels, we inadvertently duplicated the image of the spiral ligament in the MD+LPS group (Figure 4E) with that of the Vehicle group due to an error in image labeling. The corrected Figure 4 appears below.

**Figure 4:**
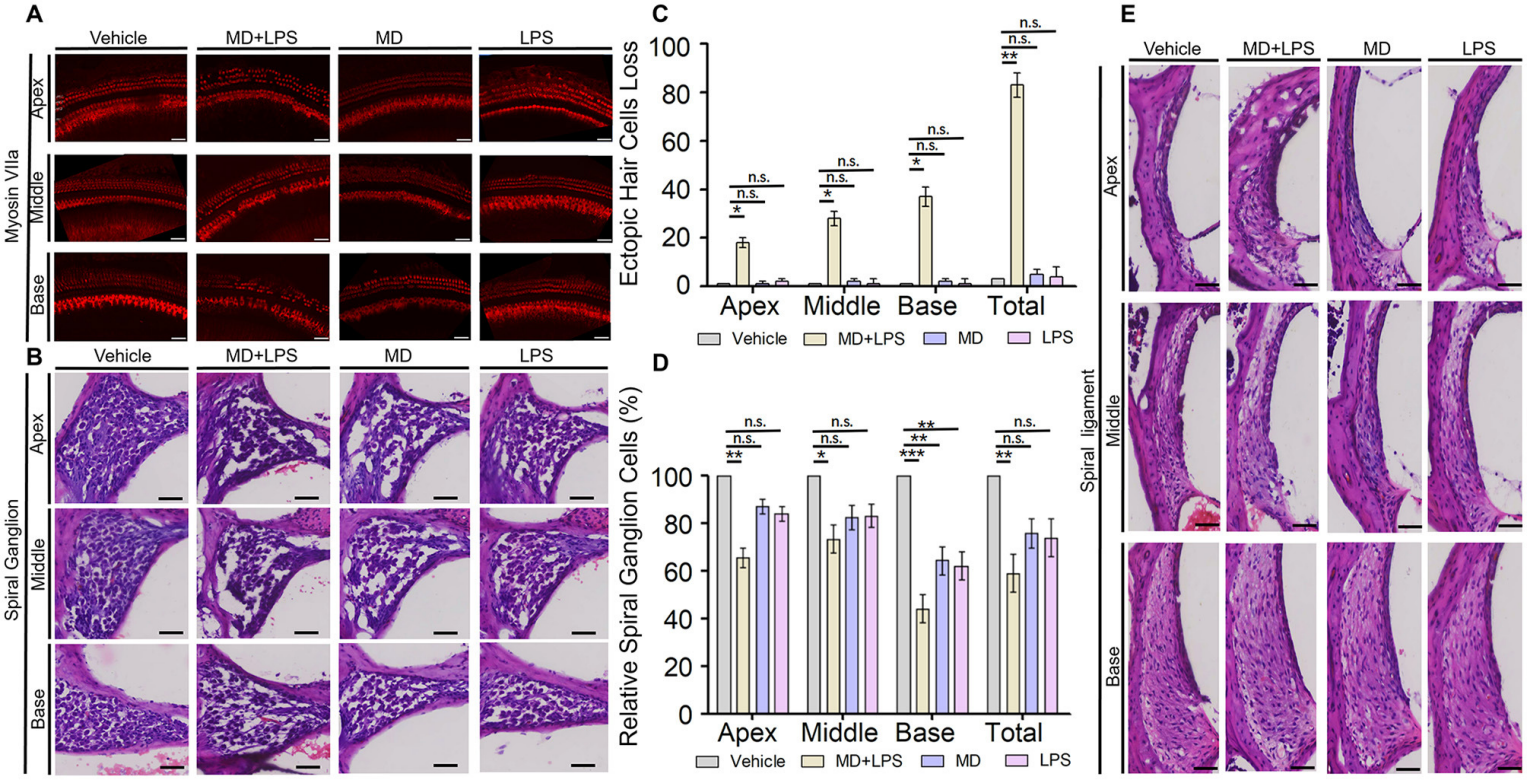
The combination of MD and LPS induces the loss of HCs and spiral ganglion cells. **(A)** Immunolabeling of the HC marker myosin VIIa. Confocal microscopy was employed to capture images of whole mounts of HCs in the cochlea (three mice per subgroup). *n* = 3 for each condition. Scale bar: 50 μm. **(B)** Representative H&E staining images of spiral ganglion cells in the cochlea for each group at 14 days post-drug administration (three mice per subgroup). *n* = 3 for each condition. Scale bar: 50 μm. **(C)** The numbers of three turns and overall ectopic HCs loss in the four groups of cochlea. *n* = 3 for each condition. Statistical significance was determined by two-way ANOVA. **(D)** Relative spiral ganglion cells of cochlea in four groups compared to the control group (%). *n* = 3 for each condition. Statistical significance was determined by two-way ANOVA. **(E)** Representative images of H&E staining displaying the vascular lines and spiral ligament of the cochlea (three mice per subgroup). *n* = 3 for each condition. Scale bar: 50 μm. ^*^p < 0.05; ^**^p < 0.01; ^***^p < 0.001; ns, not significant.

There was a mistake in Figure 6B as published. Due to oversight in image assembly, the DAPI and Merge channels for the LPS group were mistakenly replaced with corresponding Vehicle group images. While, accidental substitution of DAPI/Merge channels has no impact on the fluorescence quantification or the study's findings regarding necroptosis pathways. The corrected Figure 6 appears below.

**Figure 6:**
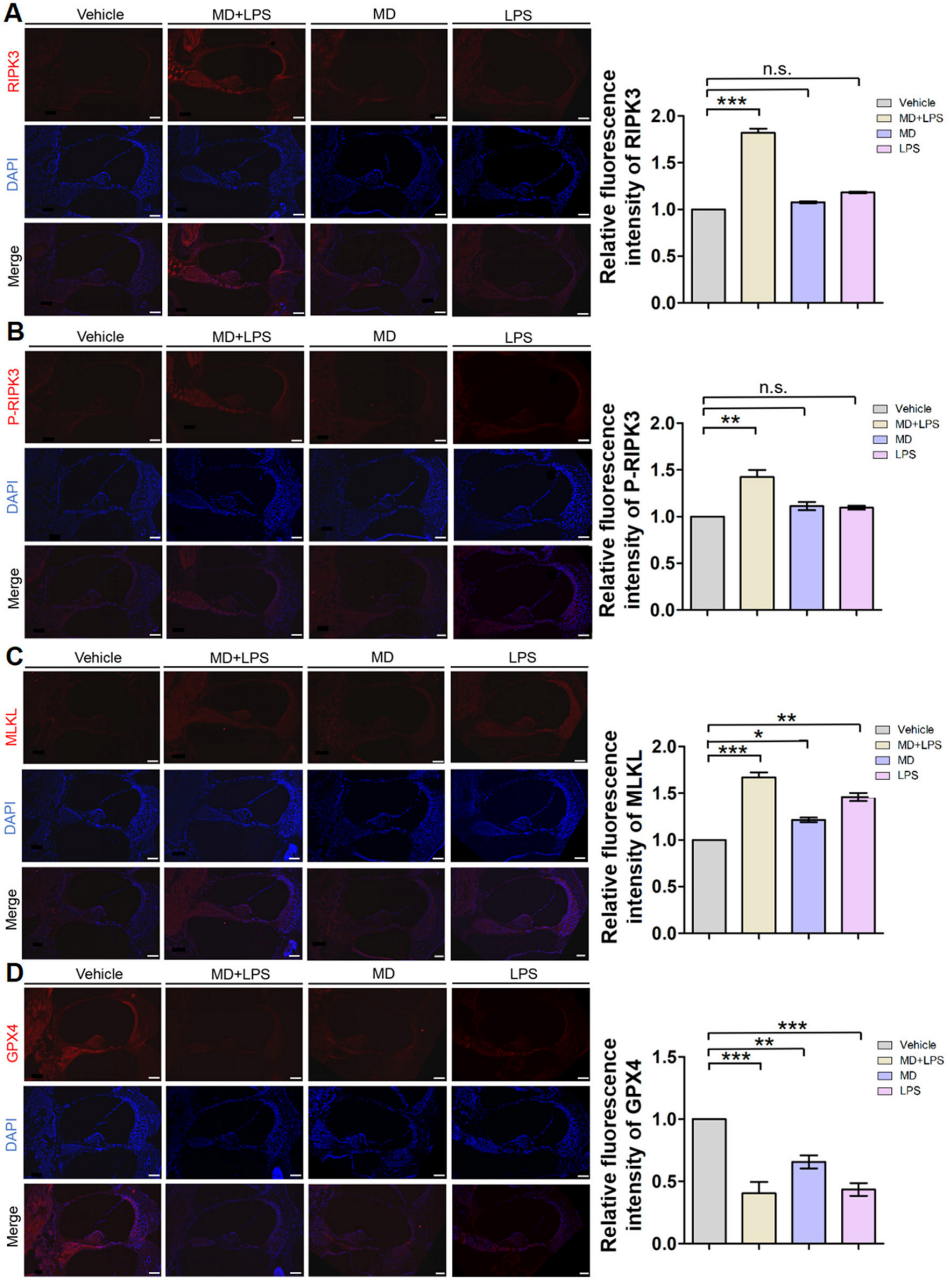
Necroptosis and ferroptosis signaling pathways are activated in cochlear sections after administration. **(A)** Representative RIPK3 immunofluorescence staining images in the cochlea. The 10 μm frozen sections of cochlea from the MD + LPS group (three mice per subgroup) show enhanced RIPK3 (red) compared to the vehicle controls. Relative fluorescence intensity of RIPK3. *n* = 3 for each condition. **(B)** Representative P-RIPK3 immunofluorescence staining images in the cochlea. The frozen sections of the cochlea in the MD + LPS group show enhanced P-RIPK3 (red) compared to the other groups. Relative fluorescence intensity of P-RIPK3. *n* = 3 for each condition. **(C)** Representative MLKL immunofluorescence staining images in the cochlea. The frozen sections of cochlea in the MD + LPS, MD and LPS groups show enhanced MLKL (red) compared to the other groups. Relative fluorescence intensity of MLKL. *n* = 3 for each condition. **(D)** Representative GPX4 immunofluorescence staining images in the cochlea. The frozen sections of cochlea in the MD + LPS, MD, and LPS groups show decreased GPX4 (red) compared to the vehicle controls. Relative fluorescence intensity of GPX4. *n* = 3 for each condition.

The original version of this article has been updated.

