# Supplementary material for: Oxidative stress and inflammation combine to exacerbate cochlear damage and sensorineural hearing loss in C57BL/6 mice
Source: Front Neurosci. 2025 Mar 5;19:1563428. doi: 10.3389/fnins.2025.1563428 (PMC11920578; doi:10.3389/fnins.2025.1563428)
Supplement: Supplementary file 1 [file Data_Sheet_1.pdf]

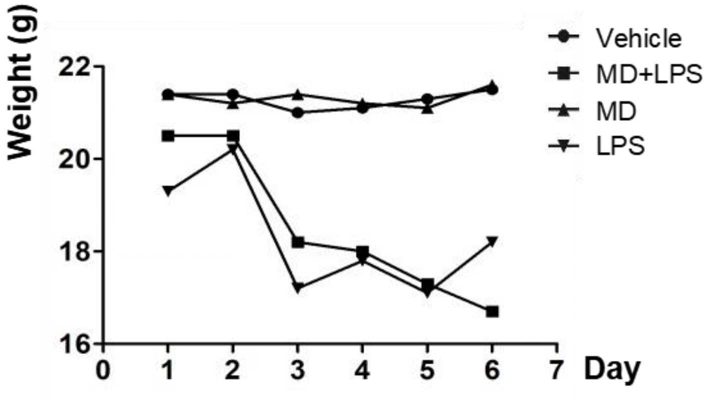

Supplement Fig. 1-1 The average weights of the four groups mice during the 6 i.p. administration

The average weight changes of mice in the MD+LPS group (n=5), MD group (n=6), LPS group (n=6), and vehicle group (n=6) during the i.p. injections.

Weight (g) during the 6 i.p. infections	Num	d1-MD	d2-LPS	d3-MD	d4-LPS	d5-MD	d6-LPS	weight loss compared to baseline
Vehicle	No.3	21.4	22.2	21.6	22	22.4	22.6	1.2
	No.4	21.1	21.9	21	21.4	21.3	21.4	0.3
	No.6	21.2	21.6	21.1	21.3	21.2	21.9	0.7
	No.17	24.2	21.3	20.8	20.6	20.9	21.1	-3.1
	No.20	19.4	19.9	20	20	20.2	20.3	0.9
	No.24	21.2	21.6	21.3	21.5	21.6	21.8	0.6
	average weight	21.4	21.4	21.0	21.1	21.3	21.5	0.1
	SD	1.54973116	0.803533862	0.546504041	0.71460945	0.731209044	0.783368794	1.596245595
MD+LPS	No.1	22.3	22.3	19.8	20	19.3	20.3	-2
	No.5	20.5	20.5	18.4	18.6	17.7	18.2	-2.3
	No.11	20.6	20.2	17.3	15.6	14.9	13.2	-7.4
	No.18 (die)	20.4	20.8	18.8	18.9	18.2	16.4	-4
	No.22	20.5	20.1	17.6	18.4	17.2	16.2	-4.3
	No.28	18.5	19	17.1	16.4	16.2	16.1	-2.4
	average weight	20.5	20.5	18.2	18.0	17.3	16.7	-3.733333333
	SD	1.20443624	1.07966044	1.032795559	1.65217029	1.544991909	2.372902583	2.033387977
MD	No.7	22.7	23.1	23	22.6	22.7	23.5	0.8
	No.8	21.1	21.1	21.3	21.9	21.2	22.1	1
	No.9	22.6	21.9	21.8	21.4	21.8	22.3	-0.3
	No.10	20	19.4	20.2	19.7	19.4	20	0
	N0.13	21.8	21.9	21.9	21.9	21.6	22	0.2
	No.15	20	19.8	20	19.8	19.9	19.7	-0.3
	average weight	21.4	21.2	21.4	21.2	21.1	21.6	0.233333333
	SD	1.20775273	1.4	1.129011367	1.199027384	1.236122971	1.461506073	0.553774924
LPS	No.16	20.8	21.3	18.5	18.9	17.9	19.2	-1.6
	No.21	21.2	22.1	19.2	19.6	18.8	20	-1.2
	No.25	18	19	16.3	17.7	16.6	18.1	0.1
	No.26	19	19.9	16.4	17	16.4	17.2	-1.8
	No.29	19.1	20.3	17.8	18	17.8	18.4	-0.7
	No.30	17.5	18.3	15.1	15.8	15.3	16.5	-1
	average weight	19.3	20.2	17.2	17.8	17.1	18.2	-1.033333333
	SD	1.47738508	1.411027994	1.543264937	1.351542329	1.264383908	1.278540835	0.683130051

Supplement Fig. 1-2 Body weight monitoring per mouse during 6-day i.p. administration regimen (g)

Following a 6-day i.p. administration regimen, one mouse subject in the MD+LPS group succumbed, likely attributable to i.p. administration intolerance. The mice in the MD+LPS group demonstrated a net body weight reduction of 3.73 ± 2 g (mean ± SD) compared to baseline measurements. In addition, comparative analysis revealed

distinct body weight patterns across the other three groups: the vehicle group exhibited a marginal mean weight gain of 0.1 ± 1.6 g, while the MD group demonstrated a slightly elevated weight increase of 0.23 ± 0.55 g. In contrast, the LPS group showed a significant net body weight reduction of 1.0 ± 0.68 g compared to baseline measurements. Comparative analysis of body weight dynamics across the four groups of mice revealed non-significant weight variations in MD-treated cohorts, contrasting with LPS-exposed groups showing overall body mass reduction.

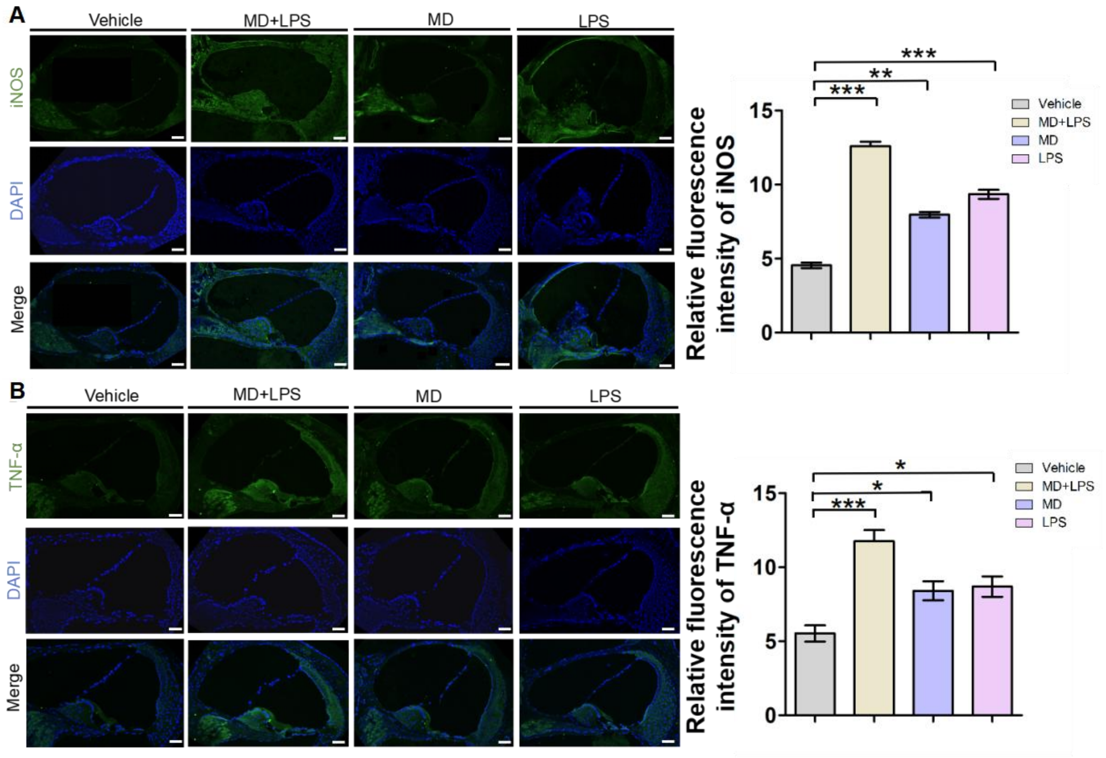

Supplement Fig. 2. Inflammatory signals are activated in cochlear sections after administration.

(A) Representative iNOS immunofluorescence staining images in the cochlea. The 10 μm frozen sections of cochlea from the MD+LPS, MD, and LPS groups (three rats per subgroup) show enhanced iNOS (green) compared to the control mice. Relative fluroscence intensity of iNOS. n = 3 for each condition.

(B) Representative TNF-α immunofluorescence staining images in the cochlea. The frozen sections of the cochlea in the other three groups show enhanced TNF-α (green) compared the control group. Relative fluroscence intensity of TNF-α. n = 3 for each condition.

The pictures above were taken from the base turn of the cochlea. Sections were counterstained with DAPI (blue) to highlight the nuclei. Scale bar: 100 μm. Statistical significance was determined by one-way ANOVA. * $p < 0.05$; ** $p < 0.01$; *** $p < 0.001$; ns: not significant. DAPI, 4’,6-diamidino-2-phenylindole

Supplement Table-1 ABR thresholds of mice in the four groups prior to administration

Group/ABR frequency	8 kHz	16 kHz	24 kHz	32 kHz
Vehicle	15.83 ± 3.76	14.17 ± 7.36	32.50 ± 4.18	39.17 ± 5.85
MD+LPS	10.00 ± 4.47	8.00 ± 4.00	28.00 ± 4.00	37.00 ± 5.10
MD	18.33 ± 4.08	14.17 ± 3.76	34.17 ± 3.76	45.00 ± 7.75
LPS	15.83 ± 4.92	14.17 ± 4.92	34.17 ± 8.01	44.17 ±8.61

The data are shown as the means ± SD of each group, with the unit being dB SPL

Supplement Table-2 ABR thresholds of mice in the four groups on day 1 post-administration

Group/ABR frequency	8 kHz	16 kHz	24 kHz	32 kHz
Vehicle	18.33 ± 2.58	13.33 ± 2.58	27.50 ± 5.24	34.17 ± 4.92
MD+LPS	35.00 ± 19.69	46.00 ± 13.87	61.00 ± 14.75	67.00 ± 15.65
MD	19.17 ± 6.64	11.67 ± 6.05	27.50 ± 5.24	37.50 ± 10.37
LPS	22.50± 5.24	21.67 ± 8.76	34.17 ± 8.61	39.17 ±12.41

The data are shown as the means ± SD of each group, with the unit being dB SPL

Supplement Table-3 ABR thresholds of mice in the four groups on day 7 post-administration

Group/ABR frequency	8 kHz	16 kHz	24 kHz	32 kHz
Vehicle	18.33 ± 4.08	16.67 ± 5.16	25.83 ± 4.91	35.83 ± 4.92
MD+LPS	39.00 ± 25.35	49.00 ± 21.01	60.00 ± 18.37	65.00 ± 17.32
MD	13.33 ± 5.16	14.17 ± 3.77	24.17 ± 7.36	31.67 ± 6.06
LPS	18.33 ± 7.52	19.17 ± 12.01	31.67 ± 4.08	35.00 ± 12.65

The data are shown as the means ± SD of each group, with the unit being dB SPL

Supplement Table-4 ABR thresholds of mice in the four groups on day 14 post-administration

Group/ABR frequency	8 kHz	16 kHz	24 kHz	32 kHz
Vehicle	20.00 ± 3.16	7.50 ± 4.18	23.33 ± 5.16	41.67 ± 6.05
MD+LPS	32.00 ± 24.14	36.00 ± 24.08	54.00 ± 20.74	74.00 ± 8.94
MD	17.50 ± 6.89	10.00 ± 3.16	27.50 ± 4.18	47.50 ± 4.18
LPS	20.83 ± 9.70	15.00 ± 10.95	30.83 ± 9.70	51.67 ± 12.51

The data are shown as the means ± SD of each group, with the unit being dB SPL
